# Pediatric bronchial ulcers induced by ibuprofen overdose: a case report combined with network toxicology to decipher potential mechanisms

**DOI:** 10.3389/fphar.2026.1892875

**Published:** 2026-09-10

**Authors:** Yixuan Huang, Guosheng Qiu, Qiongyan Li, Bin Xiong, Yiyu Chen

**Affiliations:** 1 Department of Pharmacy, The First Affiliated Hospital of Guangxi Medical University, Nanning, China; 2 Department of PICU, The First Affiliated Hospital of Guangxi Medical University, Nanning, China

**Keywords:** bronchial mucosal injury, bronchial ulcer, child, ibuprofen, network toxicology

## Abstract

**Background:**

Ibuprofen overdose is commonly associated with gastrointestinal and renal toxicity; however, severe respiratory complications such as bronchial ulceration are rarely reported, and the underlying molecular mechanisms are poorly understood.

**Objective:**

To investigate the potential molecular mechanisms of bronchial ulceration induced by an extreme ibuprofen overdose in a pediatric patient by integrating clinical observation with network toxicology-based computational predictions.

**Methods:**

We report a pediatric case of bronchial ulceration following a 360 mg/kg ibuprofen overdose, supplemented by a systematic literature review. Network toxicology (shared target identification, PPI network, GO/KEGG enrichment), molecular docking, and ADME prediction were performed.

**Results:**

Bronchoscopy on day 14 revealed multiple well-demarcated ulcers with pseudomembranes and markedly elevated IL-6 (1050.0 pg/mL). Network toxicology identified 120 shared targets, with TNF-α, IL-6, and IL-1β as hub genes. KEGG enrichment computationally predicted involvement of the IL-17 and AGE-RAGE pathways. Molecular docking confirmed strong binding affinities (<−5 kcal/mol), while ADME predictions indicated high BBB permeability and DILI risk.

**Conclusion:**

In conclusion, extreme ibuprofen overdose can induce bronchial ulceration in children, as illustrated by this rare case. In silico predictions suggest that the toxic surge of free ibuprofen may perturb inflammatory homeostasis via potential off-target interactions, computationally implicating IL-17/AGE-RAGE axes, leading to structural epithelial necrosis. While requiring further validation, this nuances classical paradigms and alerts clinicians to non-spasmodic airway injury in pediatric overdose.

## 1 Introduction

Non-steroidal anti-inflammatory drugs (NSAIDs) are widely prescribed in clinical practice for antipyretic, analgesic, and anti-inflammatory indications in both adult and pediatric populations. Their therapeutic effects primarily arise from the inhibition of cyclooxygenase-2 (COX-2), whereas most adverse reactions are linked to the suppression of cyclooxygenase-1 (COX-1) (Chou et al., 2006). Ibuprofen is a classic non-selective COX inhibitor, meaning that at conventional therapeutic doses, it predominantly blocks COX-2 to exert its efficacy, while partially inhibiting COX-1, which is responsible for maintaining mucosal integrity in the gastrointestinal tract and, importantly, the respiratory epithelium via prostaglandin E2 (PGE2) synthesis (Rainsford, 2009; Machado-Carvalho et al., 2014). However, in overdose scenarios, this non-selective inhibition is drastically amplified; the overwhelming suppression of COX-1 depletes cytoprotective prostaglandins, disrupting the delicate balance between mucosal protection and damage, and potentially extending its toxicity beyond the typical gastrointestinal (Rakovchik and Fein, 2016; Bindu et al., 2020; Panchal and Prince Sabina, 2023) and renal systems to the respiratory mucosa—a complication that remains exceedingly rare but life-threatening.

Notably, children possess immature hepatic and renal metabolic systems, resulting in a lower tolerance threshold and prolonged drug half-life compared to adults (Rainsford, 2009; Rakovchik and Fein, 2016). At conventional therapeutic doses, the risk of ibuprofen-induced severe respiratory injury is negligible (Chung and Tat, 2016). However, in cases of accidental ingestion or improper administration leading to massive overdose, the pharmacological effects of ibuprofen may undergo non-selective amplification, triggering rare complications such as bronchial ulceration. Nevertheless, the direct causal relationship between an extreme single overdose and subsequent bronchial lesions in a specific pediatric patient is inherently difficult to establish solely through clinical observation, as multiple confounding variables—including concurrent infections, nutritional status, pre-existing subclinical airway inflammation, drug interactions, and individual variations in drug metabolism and half-life—may also contribute to or exacerbate mucosal injury.

Currently, research on NSAID-related respiratory adverse reactions (such as aspirin-exacerbated respiratory disease [AERD]) has been predominantly conducted in adult populations, with scarce data available in children. Emerging evidence indicates that NSAIDs may impair mucosal repair capacity by inhibiting the COX pathway and reducing PGE2 production, but the underlying molecular mechanisms remain poorly understood (Villegas et al., 2004; Machado-Carvalho et al., 2014). Furthermore, traditional case reports are largely limited to descriptions of clinical manifestations, lacking systematic exploration of the complex biological networks that govern drug-target interactions, which significantly hinders a comprehensive mechanistic understanding. Given these gaps, we must acknowledge that a single-case observation—while clinically valuable—is insufficient to conclusively establish a toxicological profile without rigorous mechanistic investigations.

To address these knowledge gaps and generate testable mechanistic hypotheses, we adopt a combined strategy of clinical case reporting and network toxicology. Network toxicology is a computational prediction tool that integrates multi-source biological data to construct “drug-target-disease” interaction networks (Raies and Bajic, 2016; Uesawa, 2024); however, it is important to emphasize that its findings are predictive rather than definitive, and in the absence of subsequent *in vivo* or *in vitro* validation, these pathway enrichments should be interpreted as computational conjectures to guide future experimental research. Through this integrated approach, the specific objectives of this study are threefold: (1) to present a detailed clinical course of a pediatric patient who developed bronchial ulcers following a massive ibuprofen overdose, highlighting the clinical alert; (2) to apply network toxicology to computationally predict the shared direct targets and signaling pathways (e.g., IL-17 and AGE-RAGE) through which ultra-high-dose ibuprofen might act on bronchial mucosa, providing a mechanistic hypothesis for the observed injury; and (3) to offer a theoretical foundation for clinical monitoring and early intervention of drug-induced airway injury in children, while clearly framing these pathway findings as predictive and in need of further experimental validation.

## 2 Materials and methods

### 2.1 Case data collection

This study reports a single case of a 13-year-old female patient who was admitted to the Pediatric Intensive Care Unit (PICU) of our hospital following accidental ingestion of a massive dose of ibuprofen. Clinical data were retrospectively collected from the medical records, including the time of ibuprofen ingestion, serial measurements of inflammatory markers (C-reactive protein [CRP], procalcitonin [PCT], and interleukin-6 [IL-6]) on days 9 and 14 post-ingestion, and bronchoscopic findings with photographic documentation of airway mucosal lesions at the corresponding time points. The extent and distribution of bronchial lesions were thoroughly documented for analysis. Written informed consent for publication was obtained from the patient’s legal guardian. The study was approved by the Ethics Committee of the First Affiliated Hospital of Guangxi Medical University (Approval No. 2026-E0203, dated 10 March 2026) and conducted in accordance with the Declaration of Helsinki.

### 2.2 Literature search and selection

A systematic literature search was performed across PubMed, Embase, Web of Science, Reactions Weekly (via ProQuest Health and Medical Collection), and the Cochrane Library, to identify published pediatric cases of bronchial mucosal injury (BMI) associated with ibuprofen exposure, using keywords including “ibuprofen”, “bronchial mucosal injury”, and “child”. Retrieved articles were screened against predefined inclusion and exclusion criteria to support the subsequent network toxicology analysis.

### 2.3 Network toxicology

Potential targets of ibuprofen were obtained from four databases: SwissTargetPrediction, AES, PharmMapper, and CTDbase. Targets associated with bronchial mucosal injury were retrieved from the GeneCards and OMIM databases. Intersecting (common) targets of the drug and disease were identified using the online tool VENNY 2.1. Protein-protein interaction (PPI) data for these common targets were acquired from the STRING database with a minimum required interaction score of 0.4 (medium confidence), then imported into Cytoscape V3.10.2 for PPI network construction and visualization. The top 10 hub genes were screened based on network topology parameters. Gene Ontology (GO) and Kyoto Encyclopedia of Genes and Genomes (KEGG) pathway enrichment analyses were performed using the DAVID database. Finally, a “Drug-targets-pathways-disease” interaction network was constructed using Cytoscape V3.10.2. All network toxicology analyses are computational predictions intended to generate mechanistic hypotheses and do not substitute for experimental validation.

### 2.4 Molecular docking

To validate the binding interactions between ibuprofen and the core target proteins—TNF-α, IL-6, and IL-1β, which were screened as hub genes from the PPI network—as well as its direct pharmacological targets COX-1 and COX-2, molecular docking was performed. The inclusion of COX-1 and COX-2 was based on the fact that ibuprofen is a non-selective cyclooxygenase inhibitor, which may underlie its propensity to induce mucosal damage and ulceration.

The three-dimensional (3D) structure of ibuprofen was generated and subjected to energy minimization using Open Babel software with the MMFF94 force field. The crystal structures of the target proteins were retrieved from the Protein Data Bank (PDB) with the following accession codes: TNF-α (PDB ID: 6Q00), IL-6 (PDB ID: 8DPV), IL-1β (PDB ID: 8RYS), COX-1 (PDB ID: 6Y3C), and COX-2 (PDB ID: 4PH9). Protein structures were prepared by removing water molecules, adding polar hydrogen atoms, and assigning Kollman charges using AutoDockTools. Molecular docking was performed using AutoDock, with the grid box centered at the binding site of each target protein. The binding affinities were calculated as docking scores (kcal/mol), and the binding conformations with the lowest free energy were selected for further analysis. Visualization of protein-ligand interactions was performed using PyMOL.

### 2.5 ADME prediction

To evaluate the pharmacokinetic properties and drug-likeness of ibuprofen, absorption, distribution, metabolism, and excretion (ADME) analyses were performed using the SwissADME and ADMETlab 3.0 web servers. The evaluated parameters included gastrointestinal absorption, blood-brain barrier permeability, plasma protein binding, and cytochrome P450 (CYP) enzyme inhibition profiles (CYP1A2, CYP2C9, CYP2C19, CYP2D6, and CYP3A4).

## 3 Results

### 3.1 Case report

A 13-year-old female patient (height 155 cm, weight 40 kg, BMI 16.6 kg/m^2^) with no history of allergy or asthma was admitted to our PICU after ingesting a single massive dose of ibuprofen (approximately 360 mg/kg). Prior to transfer, she had received gastric lavage and respiratory support at an external hospital, and was subsequently referred to our institution for anti-infective therapy, emergency exploratory laparotomy, and other symptomatic treatments. Admission diagnoses included drug intoxication, septic shock, sepsis, suspected acute peritonitis, pulmonary hemorrhage, severe pneumonia, gastrointestinal bleeding, respiratory failure, metabolic acidosis, coma, coagulation dysfunction, thrombocytopenia, moderate anemia, and electrolyte imbalances (hyponatremia, hypocalcemia, and hypochloremia).

On day 9 post-ingestion, laboratory tests revealed the following inflammatory marker levels: CRP 83.77 mg/L, PCT 9.74 ng/mL, and IL-6 169.0 pg/mL. Flexible bronchoscopy revealed mild hyperemia of the distal tracheal mucosa, with scattered flushed lesions on the wall of the right lower basal bronchus and mild hyperemia of the left upper lobe mucosa accompanied by a small amount of viscous secretions. The left lower lobe segments contained abundant thin secretions without mucus plugs, which were easily removed by negative pressure suction, and the mucosa was prone to bleeding. After bronchoalveolar lavage, light red, slightly turbid lavage fluid was retrieved; bacterial culture of the fluid yielded a small amount of Oribacterium species. A small amount of oozing blood was noted in the lingular segment of the left upper lobe.

**FIGURE 1:**
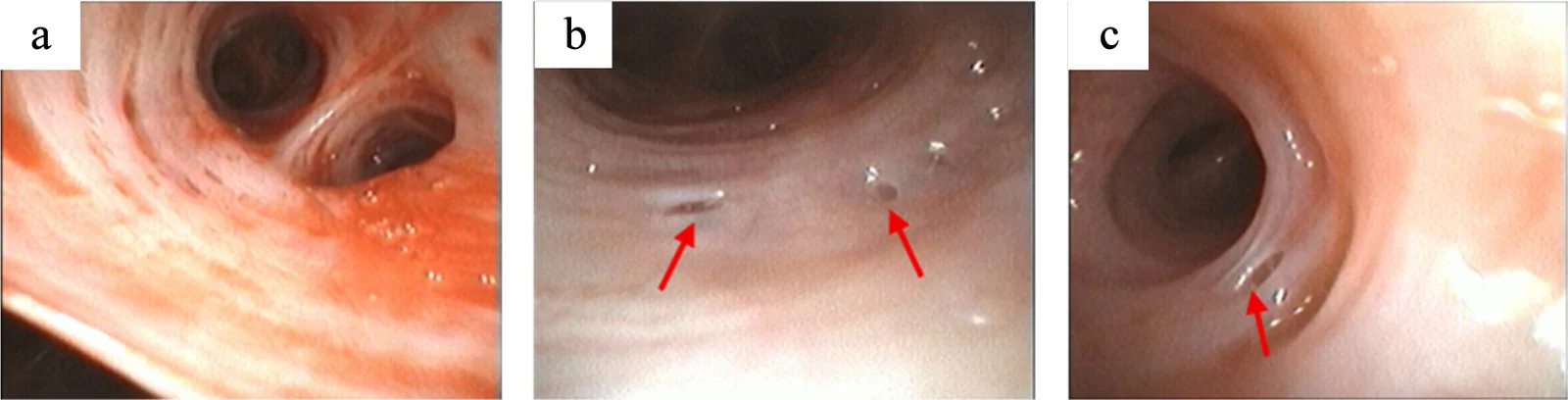
Flexible bronchoscopy findings in a child following ibuprofen overdose. **(a)** Hyperemic tracheal mucosa with multiple visible ulcers. **(b,c)** Focal bronchial mucosa presenting as smooth and pale, with well-demarcated multiple ulcers; milky-white pseudomembranes adhere to the surface of some ulcers (indicated by red arrows).

On day 14 post-ingestion, inflammatory marker levels fluctuated, with CRP rising to 142.5 mg/L, PCT decreasing to 6.46 ng/mL, and IL-6 markedly elevated to 1050.0 pg/mL. Flexible bronchoscopy showed tracheal mucosal hyperemia. Bloody viscous sputum was observed in the B6 segment of the right lung without active bleeding, while yellow-white secretions were present in the B7 segment. Notably, multiple well-demarcated ulcers were identified in the following locations: the B3 segment of the right upper lobe, the right middle lobe, the B5 segment of the right middle lobe, the B10 segment of the right lower lobe, the 4o–5o’clock direction of the left upper lobe orifice, the B3 segment of the left upper lobe proper, and the B9 segment of the left lower lobe. Some of these ulcers were covered with milky white pseudomembrane. Yellow-white turbid lavage fluid was obtained from the B6 and B7 segments of the right lung; however, bacterial smears showed no bacteria, and general bacterial and fungal cultures yielded no growth. See Figure 1 for details.

### 3.2 Literature review analysis

No reports of ibuprofen-induced bronchial mucosal injury in children were retrieved in our systematic literature search. However, Kudo H et al. (Kudo et al., 2025). Reported a 60-year-old female patient who developed toxic epidermal necrolysis (TEN) 2 days after ibuprofen administration, followed by bronchiolitis obliterans (BO) characterized by treatment-resistant beading changes, irregular bronchial walls, and bronchiectasis.

### 3.3 Network toxicology analysis

#### 3.3.1 Target collection and construction and analysis of PPI network

**FIGURE 2:**
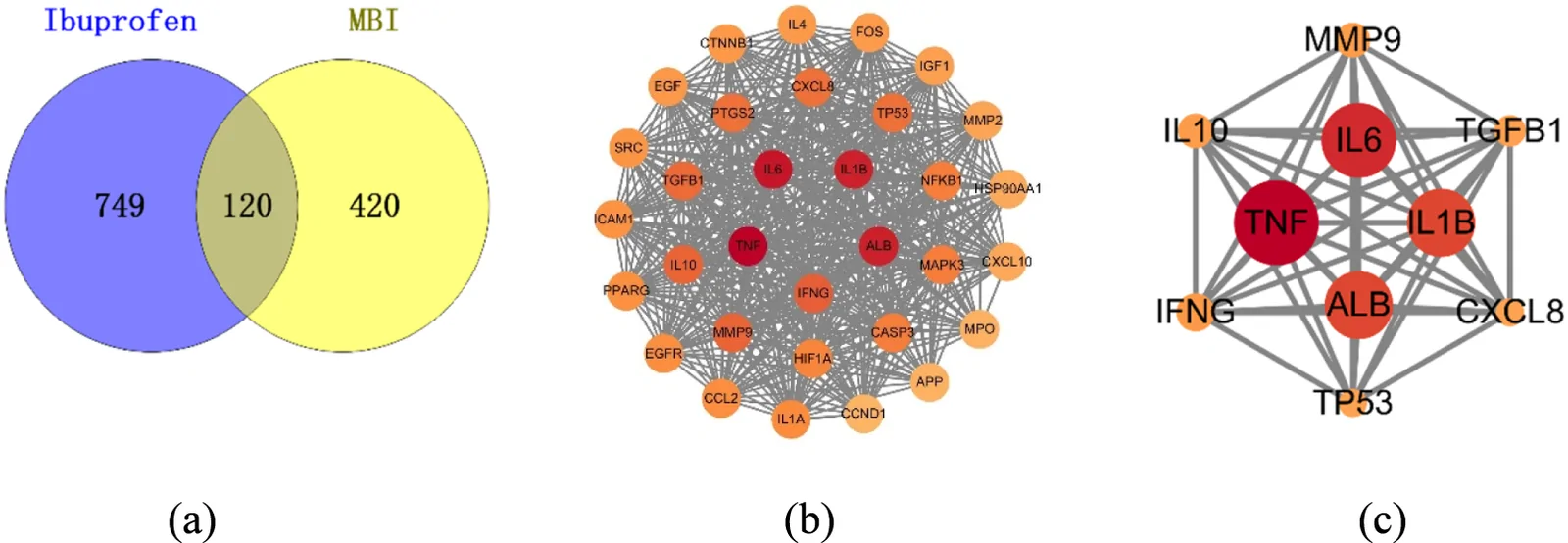
Network toxicology prediction of pathogenic targets for ibuprofen-induced BMI and construction of PPI network. **(a)** Venn diagram of targets associated with ibuprofen and BMI. The overlapping region of the blue and yellow circles denotes shared targets. **(b)** Three targets with degree values exceeding the mean in ibuprofen-induced BMI. A darker color in the figure indicates greater functional importance of the corresponding target. **(c)** Top 10 core targets for ibuprofen-induced BMI. Larger node size and darker color intensity in the figure signify higher priority and functional relevance of the target.

A total of 869 drug targets and 540 disease targets were obtained through database retrieval, with 120 overlapping genes identified as common targets. Among these, 32 targets had all three centrality measures (Betweenness, Closeness, and Degree) exceeding the average values. From these 32 targets, we further screened out the top 10 hub targets. See Figure 2 for details.

#### 3.3.2 Functional analysis of shared targets based on GO enrichment and KEGG pathway enrichment

GO enrichment analysis of ibuprofen-induced bronchial mucosal injury revealed that ibuprofen mediates mucosal damage through multiple biological processes. The top three enriched biological processes were positive regulation of transcription by RNA polymerase II, positive regulation of gene expression, and positive regulation of DNA-templated transcription. In terms of cellular components, the shared targets were mainly localized to the extracellular space, extracellular region, and protein complexes. For molecular functions, the three most significantly enriched categories were protein binding, identical protein binding, and enzyme binding.

**FIGURE 3:**
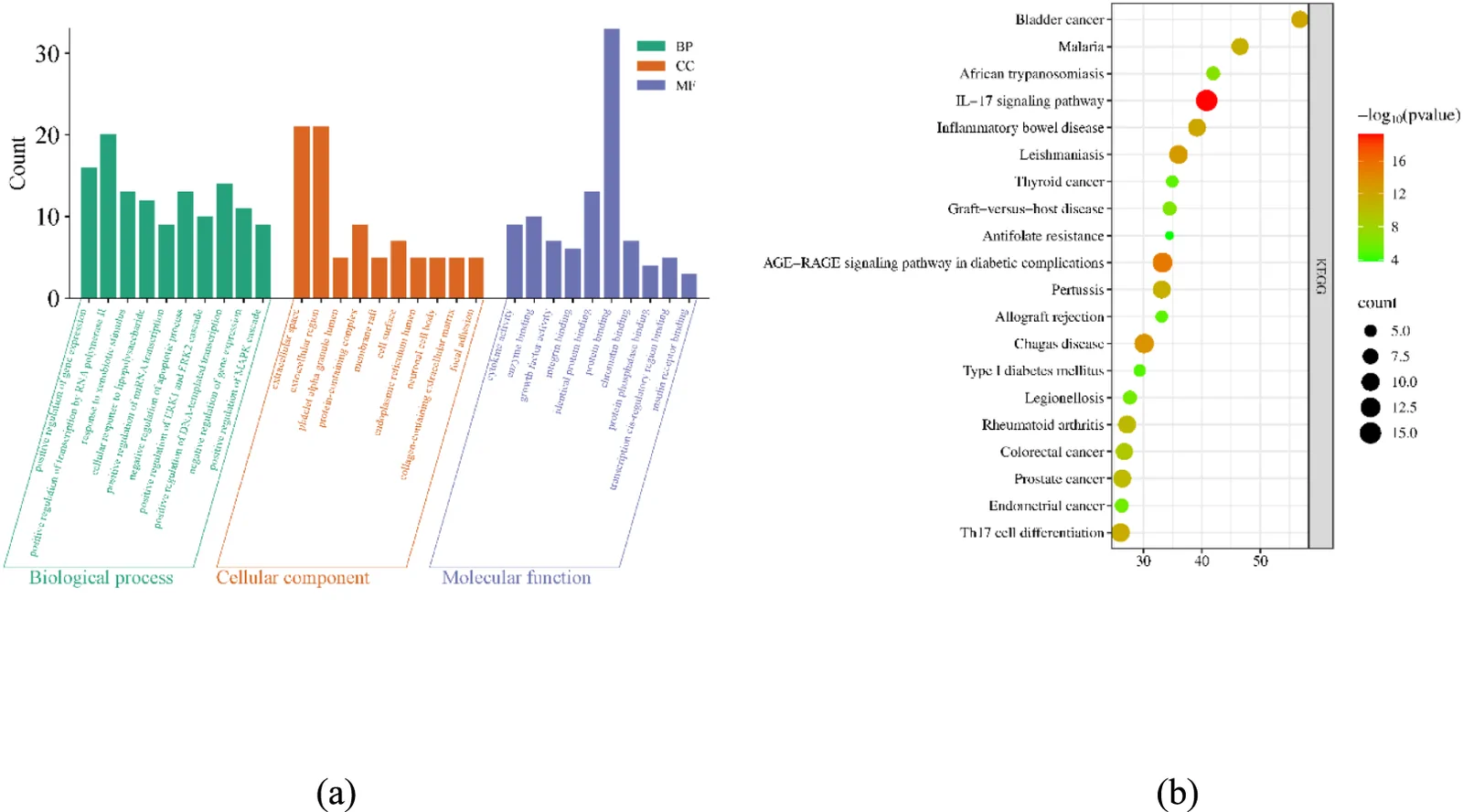
Functional enrichment analysis of shared targets. **(a)** GO enrichment bar plot (top 10 terms for Biological Process, Cellular Component, and Molecular Function). **(b)** KEGG pathway enrichment bubble plot (top 20 pathways). The node size represented the number of targets annotated to the term. The color of nodes represented the p value.

For KEGG pathway enrichment analysis of ibuprofen-induced bronchial mucosal injury, the top 20 representative pathways were selected for visualization as a bubble plot, ranked by P-value and gene count. The results showed that ibuprofen primarily acts on pathways including the IL-17 signaling pathway, AGE-RAGE signaling pathway in diabetic complications, Chagas disease, Leishmaniasis, and Th17 cell differentiation. See Figure 3 for details.

#### 3.3.3 Drug-target-pathway-disease network

**FIGURE 4:**
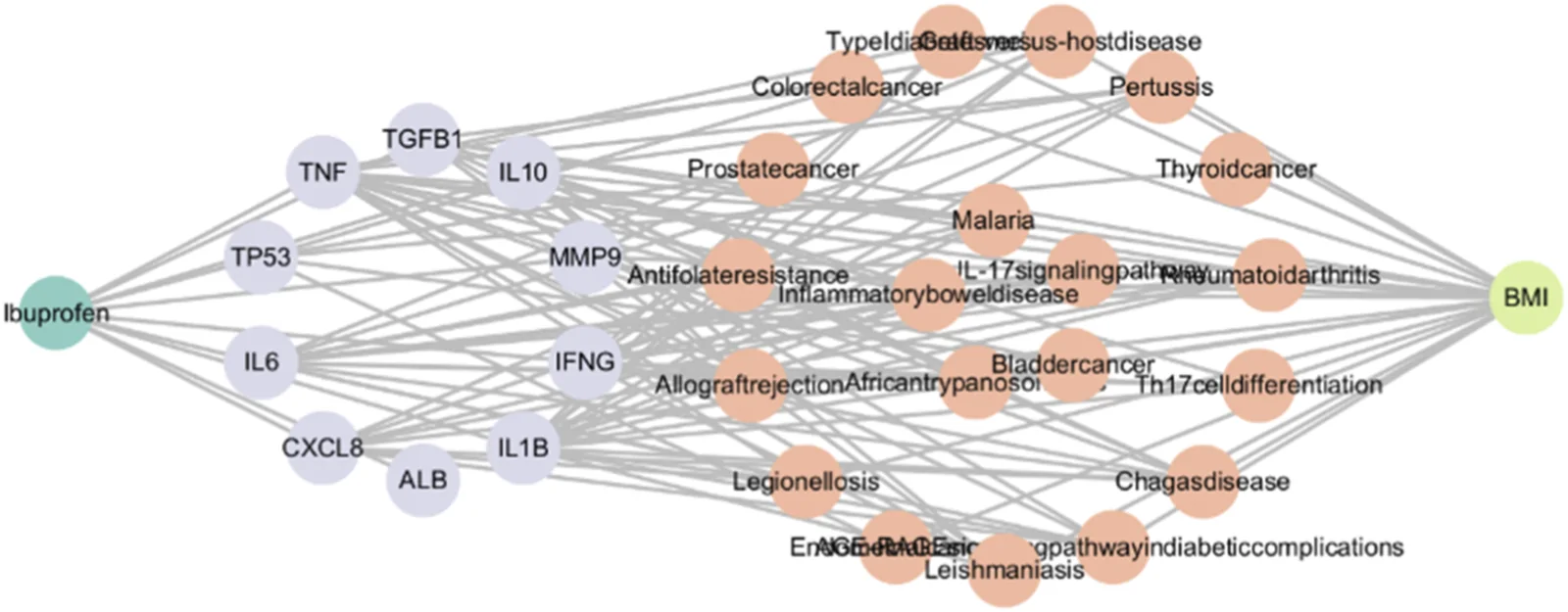
Drug-target-pathway-disease network. From left to right, the four color-coded components correspond to the drug, targets, pathways, and the disease, in that order.

We constructed a “drug-target-pathway-disease” network integrating 32 nodes, including ibuprofen, the top 10 hub genes, 20 key pathways/biological processes, and bronchial mucosal injury. The connectivity within this network reveals a multi-layered functional framework: ibuprofen acts directly on 10 hub genes; these genes are extensively involved in 20 critical biological processes and signaling pathways; ultimately, these perturbed pathways converge on the pathological outcome of bronchial mucosal injury. This network model clearly demonstrates that ibuprofen’s mucosal-damaging effect is the result of combined actions of multiple targets, which disrupt various cellular functional modules. See Figure 4 for details.

### 3.4 Molecular docking

**FIGURE 5:**
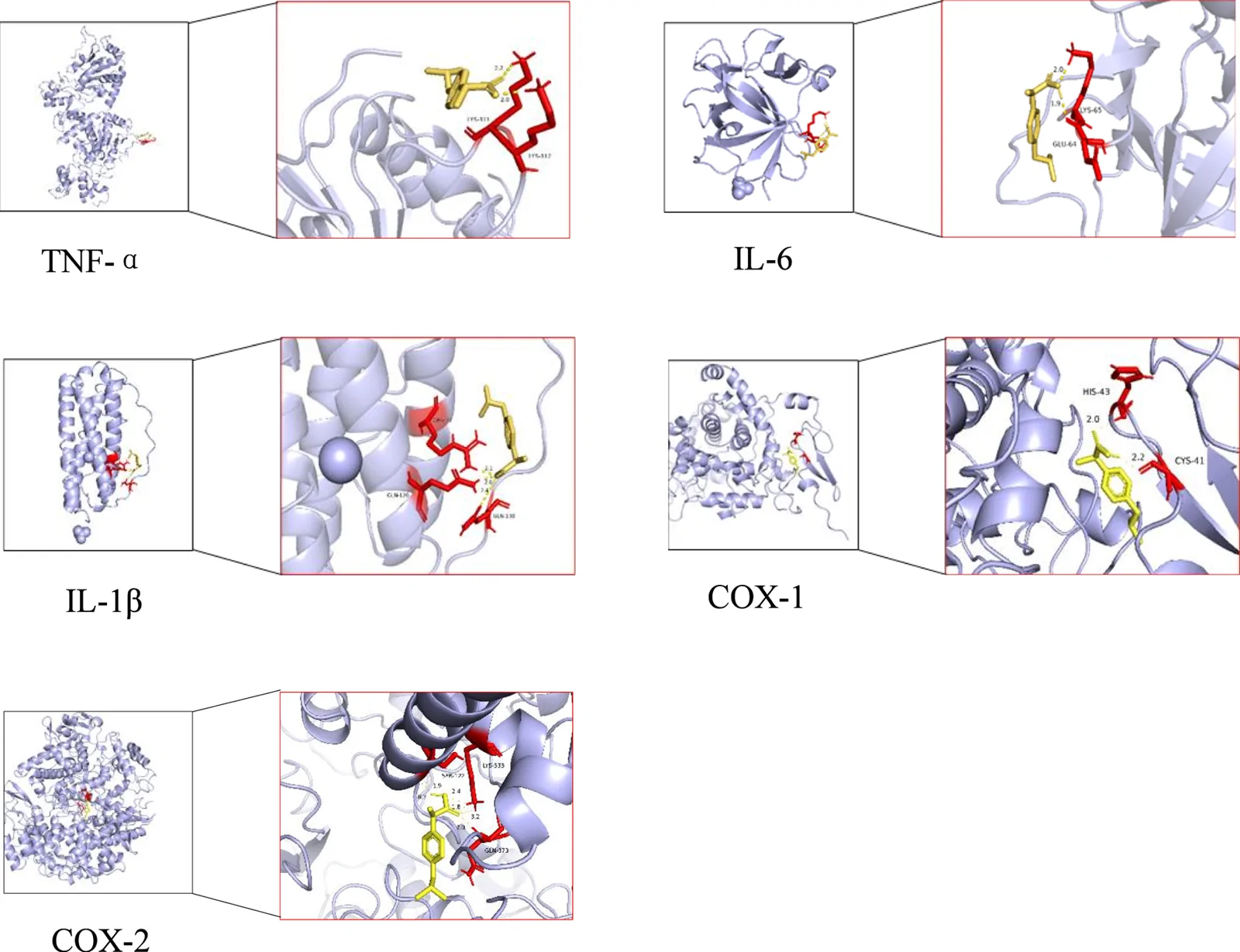
Molecular docking simulation of ibuprofen with TNF-α, IL-6, IL-1β, COX-1, and COX-2. Ibuprofen is shown in yellow, and key interacting residues are shown in red. Yellow dashed lines represent hydrogen bonds, with distances indicated in Ångströms (Å).

Molecular docking analysis was performed between ibuprofen and the three most critical core targets—TNF-α, IL-6, and IL-1β—as well as its direct pharmacological targets, COX-1 and COX-2, based on the fact that ibuprofen is a non-selective cyclooxygenase inhibitor. The docking results showed that the binding energies of ibuprofen with TNF-α, IL-6, and IL-1β were −6.33 kcal/mol, −6.02 kcal/mol, and −5.66 kcal/mol, respectively. For COX-1 and COX-2, the binding energies were −6.61 kcal/mol and −7.33 kcal/mol, respectively. All binding energy values were less than −5 kcal/mol, and at least two hydrogen bonds were formed between ibuprofen and each of these core targets, indicating stable binding affinity. Notably, ibuprofen exhibited the strongest binding affinity for COX-2 among all tested targets, consistent with its pharmacological profile as a non-selective COX inhibitor. See Figure 5, Table 1 for details.

**TABLE 1:** Molecular docking results of ibuprofen with core target proteins and cyclooxygenase enzymes.

Target protein	PDB ID	Binding affinity (kcal/mol)	Number of hydrogen bonds	Key hydrogen-bond residues
TNF-α	6Q00	−6.33	2	LYS-312, LYS-313
IL-6	8DPV	−6.02	3	ARG-77, GLN-120, GLN-130
IL-1β	8RYS	−5.66	2	GLU-64, LYS-65
COX-1	6Y3C	−6.61	2	CYS-41, HIS-43
COX-2	4PH9	−7.33	6	SER-122, GLN-373, LYS-533

### 3.5 ADME prediction results

**FIGURE 6:**
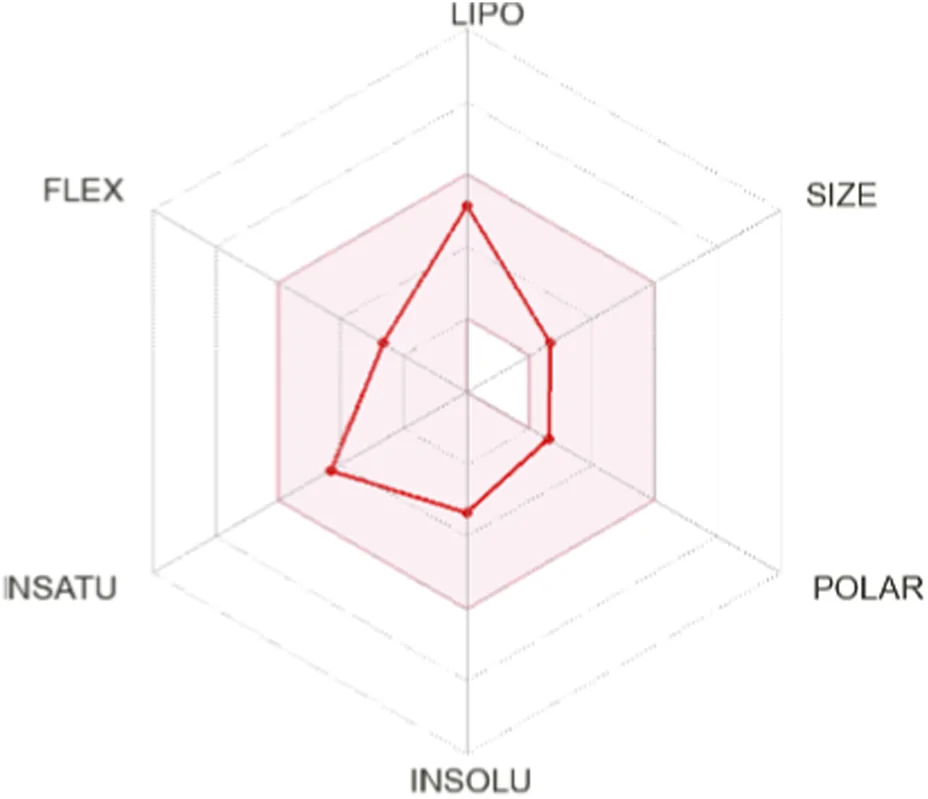
Bioavailability radar of ibuprofen. The pink shaded hexagonal area represents the optimal physicochemical space for oral bioavailability (Lipophilicity, Size, Polarity, Insolubility, Insaturation, and Flexibility). The red polygon indicates that the predicted physicochemical properties of ibuprofen fall largely within the optimal range for oral bioavailability.

The ADME analysis of ibuprofen performed using SwissADME and ADMETlab 3.0 revealed favorable drug-likeness, with no Lipinski rule violations and a high Bioavailability Score (0.85). Consistent with the evaluated parameters, the compound displayed high human gastrointestinal (GI) absorption and moderate Caco-2 permeability (−4.30 log cm/s), with no P-glycoprotein (P-gp) efflux. For distribution, the prediction indicated high plasma protein binding (PPB: 97.58%) and a notable probability of blood-brain barrier (BBB) penetration (0.61). Regarding cytochrome P450 (CYP) enzyme inhibition profiles, negligible inhibition was observed for CYP1A2, CYP2C9, CYP2C19, CYP2D6, and CYP3A4. However, the compound was predicted as a potential substrate for CYP2C19, CYP2C9, and CYP3A4. The excretion profile demonstrated a short plasma half-life (t_1_/_2_ = 1.45 h). Additionally, toxicity predictions indicated low risks of AMES mutagenicity and hERG blockade, but significant potential risks were highlighted for drug-induced liver injury (DILI) and skin sensitization. See Figure 6 and Table 2 for details.

**TABLE 2:** Summary of key ADME properties of ibuprofen.

Property	Predicted value	Remarks
GI	High	High predicted human intestinal absorption
P-gp substrate	No	Negligible efflux probability (0.126)
PPB	97.58%	High binding potential
BBB	Positive (0.61)	High probability of CNS penetration
CYP3A4 substrate	Yes (0.999)	Primarily metabolized by CYP3A4
CYP inhibition	None	No inhibition across CYP1A2, 2C9, 2C19, 2D6, 3A4
Plasma half-life (t_1/2_)	1.45 h	Short half-life
DILI	High risk (0.928)	Significant toxicity warning

## 4 Discussion

The present study documents a rare pediatric case in which a single massive overdose of ibuprofen (360 mg/kg) resulted in progressive bilateral bronchial mucosal ulcers. Serial bronchoscopy revealed a critical temporal evolution: initial mucosal hyperemia on day 9 progressed into multiple well-demarcated ulcers covered with milky-white pseudomembranes by day 14. This structural deterioration was accompanied by an overwhelming systemic inflammatory response, with IL-6 peaking at 1050.0 pg/mL. Crucially, despite profound systemic inflammation, bronchoalveolar lavage cultures consistently failed to identify pathogenic bacteria, and the anti-infective therapy did not halt the progressive airway injury. These sequential observations, particularly the progression of ulceration despite negative cultures and ongoing anti-infective therapy, argue strongly against a primary infectious etiology. However, given the concurrent septic shock and multi-organ dysfunction, we cannot definitively distinguish whether the epithelial destruction resulted from a direct cytotoxic effect of ibuprofen, an exacerbation of sepsis-induced mucosal ischemia/hypoperfusion, or a synergistic interaction. Nevertheless, the temporal progression and culture-negative profile suggest that the ibuprofen overdose likely primed the airway epithelium, rendering it hyper-susceptible to the underlying systemic inflammatory insult.

Critically, our systematic literature search across major databases retrieved no previously published pediatric cases of ibuprofen-induced bronchial mucosal injury. The only structurally comparable report was an adult case documented by Kudo et al., who described a 60-year-old female developing bronchiolitis obliterans (BO) following ibuprofen-associated toxic epidermal necrolysis (TEN) (Kudo et al., 2025). This stark pathological contrast underscores significant age-dependent heterogeneity in NSAID-induced airway toxicity (Chung and Tat, 2016). The adult case manifested as fibrotic airway remodeling driven by an adaptive immune hypersensitivity reaction (TEN), whereas our pediatric patient presented with acute epithelial necrosis and ulceration without any allergic prodrome. We hypothesize that this divergent phenotype stems from the distinct immunological and metabolic immaturity inherent to children. Rather than mounting a complex T-cell-mediated hypersensitivity response (Redwood et al., 2018), the pediatric airway epithelium appears inherently more vulnerable to direct, dose-dependent cytotoxic disruption upon massive drug accumulation, ultimately leading to structural necrosis and ulceration rather than the functional constriction or fibrosis typically observed in adults (Kudo et al., 2025). This finding expands the recognized spectrum of ibuprofen toxicity and provides a crucial clinical alert: in pediatric overdose, clinicians must actively monitor for anatomical airway destruction independent of typical bronchospasm.

This distinctive pediatric susceptibility is further reinforced by our *in silico* ADME predictions. Ibuprofen exhibited high plasma protein binding (PPB: 97.58%). At conventional therapeutic doses, this high binding limits the unbound, pharmacologically active free drug fraction. However, in a massive overdose scenario such as ours, plasma albumin binding sites become rapidly saturated, causing a dramatic surge in the free drug concentration. As ibuprofen is predicted to be a substrate for CYP3A4 with a short plasma half-life (1.45 h), its hepatic clearance capacity can be easily overwhelmed in a pediatric patient with immature metabolic systems and concomitant multi-organ failure, leading to severe toxic accumulation. This sharp rise in free, bioavailable ibuprofen may allow the drug to exceed its classical COX-selective pharmacological window and drive potent “off-target” effects on systemic and local inflammatory networks.

The molecular basis of this structural injury was computationally deciphered through network toxicology and molecular docking. While ibuprofen demonstrated stable binding affinities with its canonical targets COX-1 (−6.61 kcal/mol) and COX-2 (−7.33 kcal/mol), it unexpectedly exhibited favorable binding energies with the core pro-inflammatory cytokines TNF-α (−6.33 kcal/mol), IL-6 (−6.02 kcal/mol), and IL-1β (−5.66 kcal/mol). We postulate that at ultra-high free drug concentrations, ibuprofen physically interacts with these pivotal inflammatory mediators. Such direct, high-affinity binding could interfere with their normal metabolic clearance or disrupt their protein stability, effectively prolonging their biological activity and potentially precipitating a self-perpetuating inflammatory cascade. The KEGG enrichment analysis computationally predicted the IL-17 signaling pathway and AGE-RAGE pathway as the most prominent dysregulated networks. Mechanistically, the IL-17 axis plays a critical role in airway epithelial defense (Luo et al., 2019); it promotes the release of CXCL8 (IL-8) from epithelial cells, which facilitates robust neutrophil recruitment and directly compromises epithelial barrier integrity (Fujie et al., 2012; Honda et al., 2016; Ravi et al., 2019). In our PPI network, CXCL8 formed a central hub alongside TNF-α and IL-1β, providing a theoretical bridge connecting ibuprofen’s direct cytokine binding to the eventual disruption of the bronchial epithelium. Furthermore, the enrichment of the AGE-RAGE pathway aligns closely with our clinical observations. The patient’s profound metabolic acidosis and hyperlactatemia upon admission were clear indicators of acute mitochondrial dysfunction and extreme oxidative stress (Danhauser et al., 2015; Akar et al., 2025). Such hypermetabolic states are well-established drivers of advanced glycation end-product (AGE) formation (Gaens et al., 2013; Twarda-Clapa et al., 2022). Activation of the AGE-RAGE axis likely synergizes with the IL-17-driven inflammatory cascade, amplifying both the systemic and local immune responses, and driving the progressive mucosal injury towards irreversible necrosis characterized by pseudomembrane-covered ulcerations.

From a translational clinical perspective, our ADME toxicity warnings provide actionable insights for bedside management. The predicted high risk of drug-induced liver injury (DILI) and the notable probability of blood-brain barrier (BBB) penetration signify that severe ibuprofen overdose is a genuine multi-organ threat. Therefore, for pediatric overdose patients presenting with persistent systemic inflammation, even in the absence of initial respiratory symptoms, clinicians must maintain a high index of suspicion for delayed airway mucosal injury. Early and repeated bronchoscopic evaluation is not merely diagnostic but an essential tool for detecting occult epithelial lesions and guiding timely supportive interventions.

Despite the comprehensive computational framework, we must explicitly acknowledge the fundamental limitations of this study. The mechanistic conclusions—including the IL-17 activation and the direct cytokine binding—are entirely derived from static *in silico* bioinformatics associations and molecular docking simulations. While they present highly plausible and biologically grounded hypotheses, they inherently cannot substitute for direct *in vivo* functional validation. The actual physical binding and pathway activation must be rigorously confirmed by future animal models or *in vitro* cell-based assays. Additionally, the inherent nature of a single-case report limits the generalizability of our findings; the patient’s critical septic shock and multi-organ dysfunction certainly introduced multiple confounding inflammatory cascades (such as DAMPs, TLRs, and NLRP3 inflammasome pathways) that cannot be entirely isolated or distinguished by our current computational pipeline (Fujie et al., 2012).

In summary, this study bridges bedside clinical observations with predictive computational toxicology to propose a novel dual-mechanism model for severe pediatric ibuprofen intoxication: classical COX inhibition superimposed by an ultra-high concentration-driven “off-target” binding to core inflammatory cytokines, which may perturb the IL-17 inflammatory axis and AGE-RAGE pathway, suggesting a contribution to epithelial necrosis. Our findings highlight the critical necessity for heightened clinical vigilance regarding silent airway structural injuries in pediatric drug overdose cases and provide a strong theoretical framework to guide future experimental toxicological research.

## 5 Conclusion

This study reports a rare pediatric airway structural injury following an extreme ibuprofen overdose. Integrated computational predictions suggest that the toxic surge of unbound free drug may perturb inflammatory networks via potential off-target interactions, computationally implicating the IL-17/AGE-RAGE inflammatory axes, and may contribute to epithelial necrosis rather than typical bronchospasm. This mechanism nuances classical NSAID toxicity dogma and underscores the critical need for vigilance regarding non-spasmodic airway damage in pediatric overdose. However, these *in silico* hypotheses require further *in vivo* experimental validation. Future age-specific toxicological studies are warranted to refine pediatric medication safety interventions.

## Data Availability

The original contributions presented in the study are included in the article/supplementary material, further inquiries can be directed to the corresponding author.
